# Thyroïdectomies pratiquées sous anesthésie locale au Centre Hospitalier Universitaire d'Antananarivo

**DOI:** 10.11604/pamj.2015.21.278.7008

**Published:** 2015-08-12

**Authors:** Rex Mario Razafindrakoto, Mananjara Nandrianina Razafindranaivo, Herimalalaniaina Angelo Valisoa, Mahamad Rojovolaarivony Schammirah, Rado Randriamboavonjy

**Affiliations:** 1 Service d'Oto-Rhino-Laryngologie et de Chirurgie Cervico-Faciale, Centre Hospitalier Universitaire d'Andohatapenaka, Antananarivo, Madagascar; 2 Faculté de Médecine Humaine d'Antananarivo, Madagascar

**Keywords:** Anesthésie générale, anesthésie locale, anesthésie régionale, thyroïdectomie, general anaesthesia, local anaesthesia, regional anaesthesia, thyroidectomy

## Abstract

Menée le plus souvent sous anesthésie générale, la chirurgie thyroïdienne peut aussi être pratiquée sous anesthésie régionale ou sous anesthésie locale. Notre objectif a été de rapporter l'expérience du Centre Hospitalier Universitaire d'Antananarivo sur l'anesthésie locale dans les thyroïdectomies. La drogue employée dans notre protocole anesthésique a été le fentanyl, administré en intraveineuse directe, associé à une infiltration sous-cutanée de lidocaïne suivant la ligne d'incision. Le/la patient(e) n'a pas été intubé(e), l'oxygène étant délivré au niveau des cavités nasales. Ont été étudiés le type de chirurgie thyroïdienne, la durée de l'intervention, la satisfaction des patient(e)s vis-à-vis de la qualité de l'anesthésie, et le coût de l'intervention. Sur 567 thyroïdectomies effectuées, 51,68% (n= 293) ont été des lobo-isthmectomies gauches, 44,44% (n= 252) des lobo-isthmectomies droites, 2,82% (n= 16) des thyroïdectomies subtotales ou totales, 1,06% (n= six) des totalisations pour carcinomes thyroïdiens. La durée des interventions a varié de 30 à 90 minutes. Un total de 83,95% (n= 476) des patient(e)s a été très satisfait de la qualité de l'anesthésie et 15,87% autres (n= 90) satisfait. Les suites opératoires ont été bonnes dans la majorité des cas. Le coût d'une thyroïdectomie a été évalué à 100- 150 dollars américains. L'anesthésie locale utilisée dans les thyroïdectomies effectuées au Centre Hospitalier Universitaire d'Antananarivo est simple, rapide, d'un coût moyen, et permet d'alarmer précocement le chirurgien en cas de lésion d'un nerf laryngé inférieur.

## Introduction

Les thyroïdectomies constituent une intervention effectuée couramment par l'oto-rhino-laryngologiste (ORL) et le chirurgien cervico-facial, et sont généralement menées sous anesthésie générale (AG) [1]. La chirurgie thyroïdienne peut être également faite sous anesthésie régionale (AR) [2–4] ou sous anesthésie locale (AL) [5–9]. Notre objectif a été de rapporter notre expérience sur l'AL dans les thyroïdectomies, en la comparant avec l'AR et à l'AG, et avec les données de la littérature.

## Méthodes

Il s'agit d'une étude rétrospective menée dans le service d'ORL et de Chirurgie Cervico-faciale du Centre Hospitalier Universitaire (CHU) d'Antananarivo, Hôpital Universitaire Joseph Ravoahangy Andrianavalona, du 1er Janvier 2008 au 31 Décembre 2012. Les supports pour la collecte des données ont été les dossiers individuels des malades, les registres de consultation et ceux de compte-rendus opératoires. Ont été inclus(e)s dans l’étude tous/toutes les patient(e)s opéré(e)s de la glande thyroïde au cours de la période considérée. Ont été exclu(e)s les patient(e)s aux dossiers incomplets et inexploitables, et n'ont pas été inclus(es) les patient(e)s qui ont présenté d’énormes tuméfactions thyroïdiennes, dont la cure chirurgicale aurait dépassé deux heures, et pour lesquelles l'AG a été d'emblée préférée.

**Figure 1:**
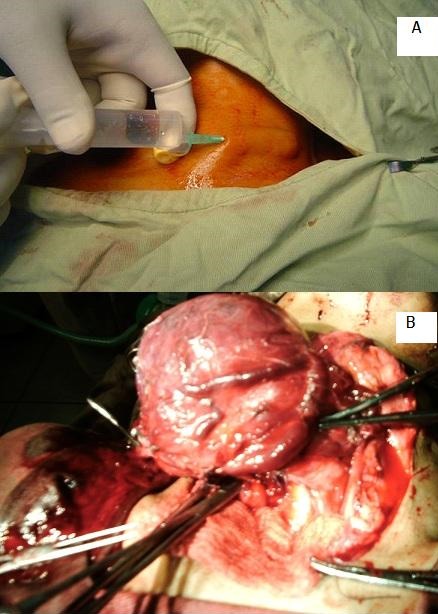
(A) ligne d'incision infiltrée avec dix millilitres de lidocaïne à 2%; (B) dissection de la glande thyroïde

Après l'interrogatoire, l'examen clinique du/de la patient(e) a inclus un examen cervical et une laryngoscopie indirecte au miroir ou par naso-laryngo-fibroscopie en pré et en postopératoire pour contrôler la mobilité des plis vocaux. La drogue employée dans notre protocole anesthésique a été le fentanyl, administrée en intraveineuse directe. Une dose de charge de 150 gammas a d'abord été administrée, puis, selon les besoins, des doses supplémentaires de 25 à 50 gammas ont été injectées, sans dépasser une dose totale de 300 gammas pour un(e) patient(e) pesant 50-60 kilogrammes. Dix millilitres de lidocaïne à 2% ont été infiltrés en sous-cutané suivant la ligne d'incision (Figure 1 A). Le/la patient(e) n'a pas été intubé(e), l'oxygène a été délivré par des «lunettes» mises en place au niveau des cavités nasales, à un débit de cinq litres par minute. La préparation anesthésique a comporté la mise en place d'une voie veineuse et la surveillance sous scope (électrocardiogramme, fréquence cardiaque, saturation en oxygène et monitoring respiratoire). La glande thyroïde a été abordée avec l'incision classique en cravate de de Kocher, allant d'un muscle sterno-cléido-mastoïdien à l'autre, et les temps opératoires ont été ceux classiques à toute thyroïdectomie (Figure 1 B) (dissection des lobes de la glande thyroïde, isthmectomie, dissection et respect des nerfs laryngés inférieurs et supéro-externes ainsi que des glandes parathyroïdes, hémostase, fermeture sur drains de Redon).

La conscience a été conservée durant toute l'intervention pendant laquelle des questions ont été posées régulièrement au/à la patient(e). Une dysphonie a été recherchée aux réponses, recherchant instantanément une atteinte des nerfs laryngés inférieurs. Le/la patient(e) a été ramené(e) directement en salle d'hospitalisation, sans passer par la salle de réveil, ni par celle de réanimation. La sortie de l'hôpital a eu lieu au deuxième jour postopératoire. Les paramètres étudiés ont été le type de chirurgie thyroïdienne, la durée de l'intervention, la satisfaction des patient(e)s vis-à-vis de la qualité de l'anesthésie ainsi que le coût de l'intervention.

## Résultats

Cinq cent soixante-sept thyroïdectomies ont été pratiquées sous AL dans notre série, représentant 26,6% de toutes les interventions ORL (n= 2.135) effectuées dans notre département durant la période d’étude. Un total de 51,68% (n= 293) des patient(e)s ont eu une lobo-isthmectomie gauche, 44,44% (n= 252) une lobo-isthmectomie droite, 2,82% (n= 16) une thyroïdectomie subtotale ou totale, 1,06% (n= six) une totalisation pour carcinome thyroïdien. La durée de l'intervention a été: entre 30 et 45 minutes pour 40,74% des patient(e)s (n= 231); entre 46 et 60 minutes pour 34,39% des patient(e)s (n= 195); entre 61 et 90 minutes pour 24,87% des patient(e)s (n= 141).

**Figure 2:**
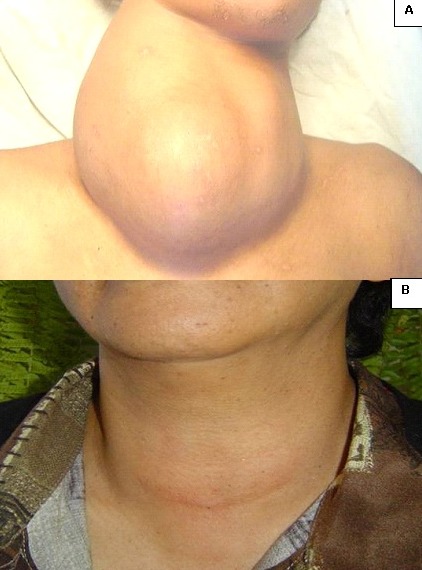
(A) gros goitre multi nodulaire opéré sous anesthésie locale; (B) aspect de la plaie opératoire six mois après

Un total de 83,95% des patient(e)s (n= 476) a déclaré avoir été très satisfait de la qualité de l'anesthésie, 15,87% (n= 90) satisfait, et aucun(e) patient(e)s (00%) ne s'est plaint(e) d'une mauvaise qualité de l'anesthésie. L'AL, initialement prévue chez une patiente (0,18%), a du être finalement convertie en AG, l'incision ayant été douloureuse. Les suites opératoires ont été bonnes, hormis 1,59% de patient(e)s (n= neuf) ayant eu une paralysie unilatérale d'un nerf laryngé inférieur, 0,35% (n= deux) une hypoparathyroïdie transitoire et 0,53% (n= trois) ayant développé une cicatrice chéloïde le long de l'incision cutanée. Il n'y a pas eu de cas de paralysie bilatérale d'un nerf laryngé inférieur, ni d'hypoparathyroïdie définitive, ni d'hématome cervical compressif. La Figure 2 A montre un gros goitre multinodulaire opéré avec succès avec notre protocole anesthésique et la qualité de la cicatrisation six mois après (Figure 2 B). Le coût d'une thyroïdectomie, incluant celui du bilan préopératoire, des consommables et des frais exigés par le CHU d'Antananarivo, a été évalué à 100- 150 dollars américains.

## Discussion

Des thyroïdectomies sont menées sous AL par d'autres équipes [5, 8, 9], notamment en Afrique, en République Centrafricaine et au Congo-Brazaville [4, 7]. L'AL n'est généralement pratiquée que sur des patient(e)s sélectionné(e)s et lorsque l'AG a été contre-indiquée [7]. La taille des séries de thyroïdectomies effectuées sous AL rapportées dans la littérature rapportée est variable: 1.025 cas par Spanknebel [8], 100 par Shukla [9], 86 par Nganga [4], 49 par Banasiewicz [7]. La nôtre a compté 567 cas, n’étant surpassée que par celle de Spanknebel.

L'AL dans les thyroïdectomies peut faire appel à une sédation induite par la kétamine [10] plutôt qu'au fentanyl comme dans notre protocole. L'infiltration par de la lidocaïne de la ligne d'incision cutanée (Figure 1) a été une particularité de notre protocole anesthésique puisque non retrouvée dans aucune des publications consultées. On peut pratiquer une AR par anesthésie épidurale avec 200 milligrammes de lidocaïne injectés entre C3 et C4 [4], sinon un bloc plexique cervical avec 20 millilitres de ropivacaïne [3]. Une AL/AR peut être convertie en AG si nécessaire [7], ce qui exige que l'AG soit disponible, comme au CHU d'Antananarivo.

L'AR permet une anesthésie durant une à deux heures [4]. L'acte opératoire ne devrait pas dépasser ces délais, et doit être effectué par des chirurgiens expérimentés [8], comme ceux exerçant au CHU d'Antananarivo. Si la thyroïdectomie devrait durer plus de deux heures et pour les trop gros goitres, il est préférable d'opter d'emblée pour l'AG. La durée moyenne des thyroïdectomies effectuées sous AL par Shukla a été de 41,6 minutes contre 74,5 minutes si l'intervention se faisait sous AG [9], alors que 426 de nos patient(e)s (75,13%) ont été opéré(e)s en moins de 60 minutes. Nos patient(e)s n'ont pas été intubé(e)s, l'oxygène ayant été délivré par des «lunettes», permettant ainsi un gain de temps par rapport à l'AG, où les malades sont intubé(e)s. Cela a procuré une diminution de la charge de travail du personnel soignant. Dans la chirurgie thyroïdienne menée sous AR, Nganga a noté une rapidité d'installation, une qualité, une innocuité et une fiabilité satisfaisantes de l'anesthésie [4]. Cinq cent soixante-six de nos patient(e)s (99,82%) ont été satisfait(e)s ou très satisfaits(e) de la qualité de notre AL. La sensation tactile est conservée, le/la patient(e) sait et sent qu'on travaille au niveau de son cou mais sans accuser de douleur.

Une meilleure qualité vocale en postopératoire peut être obtenue avec un monitoring vocal du nerf laryngé supéro-externe, dont l'atteinte est responsable d'une dysodie ou trouble de la voix chantée [6]. Dans les thyroïdectomies pratiquées sous AL, la fonction vocale peut être explorée sous vidéo-laryngo-stroboscopie en peropératoire [5], moyen d'exploration inexistant dans notre département. La voix parlée a été contrôlée durant toute la durée de nos interventions, le/la patient(e) restant vigile et le risque sur les nerfs laryngés inférieurs évaluable à tout instant. Une laryngoscopie indirecte au miroir ou une naso-laryngo-fibroscopie ont été systématiques en pré et en postopératoire chez tous/toutes nos malades.

Les patient(e)s opéré(e)s sous AL/AR se sont rétabli(e)s plus rapidement que ceux/celles opéré(e)s sous AG. La durée du séjour hospitalier, atteignant dix jours si l'intervention chirurgicale a été faite sous AG [1], a été nettement plus courte, n'ayant été que de deux jours, autant dans notre série que dans celle de Banasiewicz, opérant aussi sous AL [7]. Musa, ayant mené une étude dans des centres ruraux et semi-urbains du Nigéria, a retrouvé un coût de l'AR moins élevé que celui de l'AG [2]. Shukla, en Inde, a quantifié le coût de l'AG deux fois plus élevé que celui de l'AL [9]. Le revient financier moyennement élevé de notre protocole thérapeutique a été apprécié par nos patient(e)s aux revenus le plus souvent modestes.

## Conclusion

L'anesthésie locale utilisée dans les thyroïdectomies effectuées au Centre Hospitalier Universitaire d'Antananarivo est dotée de nombreux avantages: simplicité, rapidité, coût moyen, convertible en anesthésie générale, signes d'alarme précoces de l'atteinte d'un nerf laryngé inférieur, diminution de la charge de travail du personnel soignant. Elle pourrait être pratiquée dans des pays en développement, notamment en Afrique.
